# Association between CYP2E1 genetic polymorphisms and urinary cancer risk: a meta-analysis

**DOI:** 10.18632/oncotarget.20993

**Published:** 2017-09-18

**Authors:** Zhiqing Fang, Yun Wu, Ning Zhang

**Affiliations:** ^1^ Department of Urology, Qilu Hospital, Shandong University, Jinan, Shandong, China; ^2^ Department of Breast Surgery, Qilu Hospital, Shandong University, Jinan, Shandong, China

**Keywords:** cytochrome P4502E1, CYP2E1, polymorphism, urinary cancer, meta-analysis

## Abstract

**Objective:**

Studies investigating the contribution of Cytochrome P4502E1 (CYP2E1) polymorphisms to the etiology of urinary cancer draw inconsistent conclusions. Thus, we performed a meta-analysis to evaluate the association between CYP2E1 Rsa I/Pst I and Dra I polymorphisms and urinary cancer susceptibility.

**Materials and Methods:**

Meta-analysis based on the eligible case-control studies that assess the association of CYP2E1 Rsa I/Pst I and Dra I polymorphisms with urinary cancer was conducted. Subgroup analyses based on ethnicity and cancer type were also carried out. Odds ratios (OR) and 95% confidence intervals (95% CI) were calculated to evaluate the strength of the associations between the two polymorphisms. Funnel plot and Begg’s test were used for publication bias diagnosis.

**Results:**

We found decreased urinary cancer risk among subjects carrying CYP2E1 RsaI/PstI c1c2 + c2c2 genotype and c2 allele (OR = 0.73, 95% CI = 0.68–0.79 and OR = 0.79, 95% CI = 0.74–0.85, respectively), with 3,301 cases and 3,786 controls from 14 studies. We also observed a significant difference in c1c2 + c2c2 vs. c1c1 and c2 vs. c1 among Asians (OR = 0.68, 95% CI = 0.60–0.78 and OR = 0.75, 95% CI = 0.66–0.85, respectively). However, the meta-analysis based on 5 eligible studies showed no significant association between CYP2E1 Dra I polymorphism and urinary cancer susceptibility in either dominant model or the allele model.

**Conclusions:**

Our meta-analysis concluded that CYP2E1 Rsa I/Pst I polymorphism correlates with urinary cancers risk in Asian population; while CYP2E1 Dra I polymorphism might be not significantly associated with the urinary cancer risks. Large and well-designed studies are needed to confirm these results.

## INTRODUCTION

Cancer is a leading cause of death worldwide, which accounted for 7.6 million deaths in 2008 [1]. Prostate cancer, renal cancer and urothelial carcinoma are common types of malignancies worldwide [2]. Although the underlying mechanism of its development is largely unclear, it has been widely accepted that environmental risk factors such as cumulative cigarette smoking, alcohol consumption, certain occupational exposures, radiation and possible carcinogens including N-nitrosamines, aniline, vinyl chloride and urethane are involved in the onset of urinary cancer [3]. Nevertheless, very small fraction of individuals who exposed to the risk factors eventually become urinary cancer patients, indicating that other causes, such as genetic susceptibility, might affect the variat individual urinary cancer risk [4, 5].

Recently, a number of gene polymorphisms which were associated with urinary cancer risk have been found, and some polymorphisms located in the genes associated with carcinogen metabolism courses [6]. Human cytochrome P450 (CYP) enzymes play an important role in the metabolism of environment as well as drug chemicals. Numerous CYP enzymes could activate procarcinogens to genotoxic intermediates. An association between CYP enzyme activity and the risk to develop cancer has been revealed by phenotyping analyses. It has been demonstrated that many CYP enzymes are polymorphic owing to single nucleotide polymorphisms, gene duplications and deletions [7].

Cytochrome P4502E1 (CYP2E1) is belonged to the member of the cytochrome P450 superfamily. It is a phase I enzyme that could metabolically activate many kinds of carcinogens [8, 9]. N-nitrosamines are identified as carcinogens associated with the development of tumors of different sites [10].

CYP2E1 gene is located on chromosome10q26.3 and is consist of nine exons and eight introns. So far, over one hundred single nucleotide polymorphisms (SNPs) have been reported (http://www.ncbi.nlm.nih.gov/SNP). Though, only a few polymorphisms which might alter the enzymatic activity of CYP2E1 have drawn interest [11, 12]. Among CYP2E1 polymorphisms, rs3813867 *G > C* is associated with Pst I restriction enzyme site while rs2031920 *C > T* accounts for Rsa I restriction enzyme site. The two polymorphisms were in complete linkage disequilibrium, which results in the CYP2E1*5 haplotype and three different genotypes: homozygous of normal alleles (*c1c1, Rsa I+* /*Pst I−)*, heterozygous *(c1c2)* and homozygous after nucleotide replacement *(c2c2, Rsa I−* /*Pst I+)*[13]. Several studies demonstrated that CYP2E1 Rsa I/Pst I polymorphism is located in the promoter region of CYP2E1 gene and elevated the transcriptional activity of gene [14]. Another polymorphism (rs6413432) recognized by Dra I restriction enzyme located in intron 6, namely CYP2E1*6 polymorphism, result in three distinct genotypes: *CC, CD and DD* [15]. This polymorphism was reported to affect the transcription of the CYP2E1 gene [15] and was correlated with single strand breaks in DNA [16]. Therefore, we select these two polymorphisms to explore the association between Rsa I/Pst I and DraI and urinary cancer risk.

Recently, the associations between CYP2E1 gene and urinary cancer risk were investigated. However, the results from epidemiological studies were inconsistent and controversial [17–31]. The study conducted by Yang et al. [26] found men with CYP2E1Pst I/ Rsa I polymorphisms developed a decreased risk of prostate cancer. Additionally, the study by Choi et al. [23] confirmed that CYP2E1 Rsa I/Pst I polymorphism would confer susceptibility to bladder cancer. Yet, several studies conducted by other teams [18, 19, 24] failed to find any association between CYP2E1 Rsa I/Pst I and Dra I polymorphisms and the risk of urinary cancers. The inconsistent conclusions could be attributed to the differences in patient ethnicity or insufficient sample size. Therefore, we collected published data to study the association between CYP2E1 RsaI/PstI and DraI, and urinary cancer risk to illuminate current uncertain conclusions.

## MATERIALS AND METHODS

### Literature search strategy

We carried out a comprehensive search in the databases PubMed, Elsevier, SpringerLink and CNKI (Chinese national knowledge infrastructure) without a language limitation, covering all the papers published up to Oct 2016. The search strategy to identify all possible studies involved used combinations of the following key words: (cytochrome P4502E1 OR CYP2E1) and (polymorphism OR variant OR allele OR genotype) and (kidney OR renal OR urothelial OR transitional cell carcinoma OR bladder OR prostatic OR prostate). A cited reference search of the retrieved papers was also conducted, and further publications were also identified by retrieving the bibliographies of the retrieved papers.

### Inclusion criteria

Data from studies were included in this meta-analysis only if the study met the following criteria: (1) the study should concern the association of CYP2E1 RsaI/PstI or Dra I polymorphisms with urinary cancer risk; (2) only the observational (case-control or cohort) studies in accordance with Hardy-Weinberg equilibrium (HWE) were considered; (3) the paper must indicate the sample size, odds ratios (ORs) and their 95% confidence intervals (CI) as well as the genetic distribution or the information that can help infer the results. Accordingly, papers that could not offer the source of cases and controls or other essential information were excluded; reviews, editorial and comments were also excluded. After rigorous searching, we reviewed all the papers based on the above criteria for further analysis.

### Data extraction

Data were carefully extracted from all the eligible publications by two investigators independently according to the inclusion criteria. For each study, the following information was extracted from the study: name of first author, year of publication, region, ethnicity, gender, cancer types, matching criteria, the numbers of cases and controls with the three genotypes and genotyping methods. When the essential information was not provided in articles, every effort was made to contact the authors. In the case of conflicting evaluations, disagreements of included studies were resolved by discussion. When a consensus cannot be reached, another author was to be consulted to resolve the dispute, and then a final decision was made based on a majority of votes.

### Statistical analysis

HWE for CYP2E1 Rsa I/Pst I and Dra I polymorphisms of control groups were extracted from the original publications. In case of studies without reporting HWE status, the distributions of genotypes were tested for HWE using the Chi-square test and results with *P* value > 0.05 were considered to be in accordance with HWE. We then used Chi-square test to calculate the genotype and allele difference of CYP2E1 Rsa I/Pst I and Dra I polymorphisms in Caucasian and Asian populations. For CYP2E1 Rsa I/Pst I and Dra I polymorphisms, we only assessed the risk in the dominant model and the allele model due to few frequencies of mutated genotypes in subjects. Afterwards, we performed subgroup meta-analysis according to the status of ethnicity and cancer type. The pooled OR with 95% CI was calculated to evaluate the associations of CYP2E1 Rsa I/Pst I and Dra I polymorphisms with urinary cancer risk, using subjects with the homozygous common allele as the reference group. The significance of the overall OR was determined by the *Z*-test.

Cochran’s Chi-square based *Q* test [32] and I^2^ test [33] were adopted to evaluate possible heterogeneity in the combined studies. The *P <* 0.10 or I^2^ value ≥ 50% was considered to represent significant heterogeneity. The random effects model (the DerSimonian-Laird method), which yields wider confidence intervals, would be adopted to calculate the overall OR value if the test of heterogeneity was significant [34]; otherwise, the fixed effects model (the Mantel-Haenszel method) was adopted [35]. Publication bias is always of concern in a meta-analysis. Therefore, funnel plots were primarily drawn to evaluate potential publication bias and an asymmetric plot indicates a possible publication bias. Funnel plot asymmetry was further evaluated by Begg’s test [36] with STATA(Version 12.1).

All of the statistical analyses were performed with RevMan (Version 5.0, The Cochrane Collaboration) and STATA (Version 12.1). All the tests were two-sided and *P* value less than 0.05 was considered to be statistically significant.

## RESULTS

### Characteristics of studies

**Figure 1:**
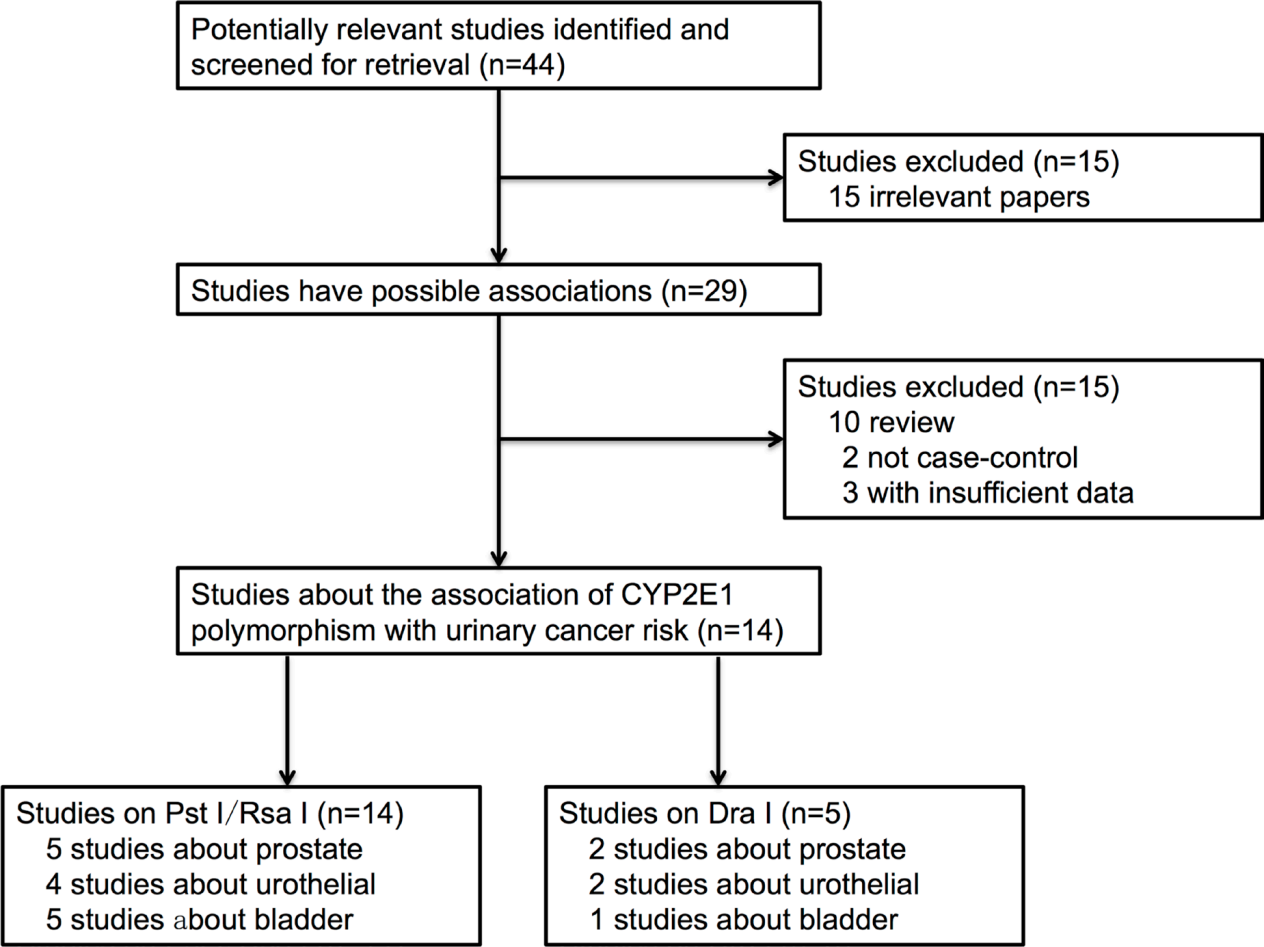
The flow diagram of search strategy in this meta-analysis

There were a total of 44 studies preliminarily identified by searching the PubMed, Elsevier, Springer Link and MEDLINE databases. After screening titles and abstracts, 29 studies were identified to be relevant. After full text searching, 15 studies were excluded. According to the eligible criteria, three studies were discarded for insufficient data (although we tried to contact the authors to query the data), two studies were discarded as being case-only studies and ten studies were discarded as being review articles. Finally, 14 studies were identified for the CYP2E1 Rsa I/Pst I polymorphism, including a total of 3,301 cases and 3,786 controls, and for the DraI polymorphism 5 studies were identified covering a total of 1,168 cases and 1,275 controls. The flow diagram of selection strategy was shown in Figure 1. The detailed characteristics of included studies were summarized in Table 1 and Table 2. Among those 14 studies, six studies [17–20, 24, 31] included Caucasians and eight studies [21–23, 26–30] included participants of Asian descent. Still, seven studies [17–20, 22, 28, 29] only recruiting male subjects focused on prostate and bladder cancer, and seven [21, 23, 24, 26, 27, 30, 31] with both male and female participant on bladder or urothelial cancer. The polymerase chain reaction restriction fragment length polymorphism (PCR-RFLP) was the most common method used to analyze the genotype frequencies of these two polymorphisms.

**Table 1:** Studies on the association between the genetic polymorphisms of the CYP2E1Rsa I/Pst I and the risk of urinary cancer included in the meta-analysis

First author	Year	Region	Ethnicity	Gender	Cancer Types	Matching criteria	Cases, n			Controls, n			Genotyping methods
							c1c1	c1c2	c2c2	c1c1	c1c2	c2c2	
Anwar [17]	1996	Egypt	Caucasian	M/F	bladder	age, smoking	22	0	0	20	1	0	PCR-RFLP
Brockmoller [18]	1996	German	Caucasian	M/F	bladder	gender, age	233	125	16	215	142	16	PCR-RFLP
Farker [19]	1998	German	Caucasian	M/F	urothelial	NA	211	13	1	289	15	0	PCR-RFLP
Farker [20]	1998	German	Caucasian	M/F	urothelial	NA	256	16	1	284	14	0	PCR-RFLP
Murata [21]	2001	Japan	Asian	M	prostate	NA	71	39	5	109	83	8	PCR-RFLP
Tsukino [22]	2002	Japan	Asian	M/F	urothelial	gender, age	93	38	6	127	77	13	PCR-RFLP
Choi [23]	2003	Korea	Asian	M	bladder	NA	124	86	4	93	89	12	PCR-RFLP
Ferreira [24]	2003	Portugal	Caucasian	M	prostate	age	91	4	0	115	8	0	PCR-RFLP
Yang [26]	2006	China	Asian	M	prostate	age	156	65	4	147	90	12	PCR-RFLP
Yang [27]	2006	China	Asian	M	prostate	age	113	50*		118	84*		PCR-RFLP
Shao [28]	2008	China	Asian	M/F	bladder	gender, age	131	62	9	170	91	11	PCR-RFLP
Wang [29]	2009	Taiwan	Asian	M/F	urothelial	age, gender	335	170	15	292	202	26	PCR-RFLP
Yang [30]	2009	China	Asian	M	prostate	age	77	32*		118	76	8	PCR-RFLP
Cantor [31]	2010	Spain	Caucasian	M	bladder	age	590	37	0	569	42	0	GoldenGate

*Frequency of genotypes “c1c2 + c2c2”.

**Table 2:** Studies on the association between the genetic polymorphisms of the CYP2E1Dra I and the risk of urinary cancer included in the meta-analysis

First author	Year	Region	Ethnicity	Gender	Cancer Types	Matching criteria	Cases, *n*			Controls, *n*			Genotyping methods
							DD	DC	CC	DD	DC	CC	
Brockmoller [18]	1996	German	Caucasian	M/F	bladder	gender, age	292	43	6	262	37	1	PCR-RFLP
Farker [19]	1998	German	Caucasian	M/F	urothelial	NA	191	33	2	262	40	2	PCR-RFLP
Farker [20]	1998	German	Caucasian	M/F	urothelial	NA	233	38	2	259	38	1	PCR-RFLP
Ferreira [24]	2003	Portugal	Caucasian	M	prostate	age	86	17**		87	36**		PCR-RFLP
Yang [26]	2006	China	Asian	M	prostate	age	145	73	7	143	91	16	PCR-RFLP

**Frequency of genotypes “DC + CC”.

### Frequency of CYP2E1 Rsa I/Pst I and Dra I polymorphisms in control population

Firstly, we evaluated the genotype and allele frequency distributions of CYP2E1 Rsa I/Pst I and Dra I polymorphisms between Caucasian and Asian control populations (Table 3). As for CYP2E1Rsa I/Pst I polymorphism, 1,730 controls of Caucasian population and 1,652 controls of Asian population were included in our analysis. With Chi-square test, we found the frequency distributions of the genotypes and alleles for CYP2E1 Rsa I/Pst I polymorphism were significantly different between Caucasian and Asian groups (both *P* value < 0.001). As for CYP2E1 Dra I polymorphism, the frequency distributions of the genotypes and alleles were also statistically significant between the Caucasian and Asian groups (both *P* value < 0.001).

**Table 3:** The genotype and allele frequencies of CYP2E1 gene Rsa I/Pst I and Dra I polymorphisms in controls from Caucasian and Asian groups

SNPs	Genotype/Allele		Caucasian		Asian		*P* value
			*n*	%	*n*	%	
Rsa I/Pst I	Genotypes*	c1c1	1492	86.24	938	56.78	
		c1c2	222	12.83	632	38.26	
		c2c2	16	0.92	82	4.96	< 0.001
		c1c2 + c2c2	238	13.76	714	43.22	< 0.001^a^
	Alleles*	c1	3206	92.66	2508	75.91	
		c2	254	7.34	796	24.09	< 0.001^b^
Dra I	Genotypes**	DD	783	86.81	143	57.20	
		DC	115	12.75	91	36.40	
		CC	4	0.44	16	6.40	< 0.001
		DC+CC	119	13.19	107	42.80	< 0.001^a^
	Alleles**	D	1681	93.18	377	75.40	
		C	123	6.82	123	24.60	< 0.001^b^

*Study by Yang [27] and Yang [30] were not included since they did not provide the frequency of c1c2 and c2c2 respectively;

**Study by Ferreira [24] was not included since it did not provide the frequency of DC and CC respectively;

^a^*P* value for the dominant models;

^b^*P* value for the allele models.

### Main results of meta-analysis

**Figure 2:**
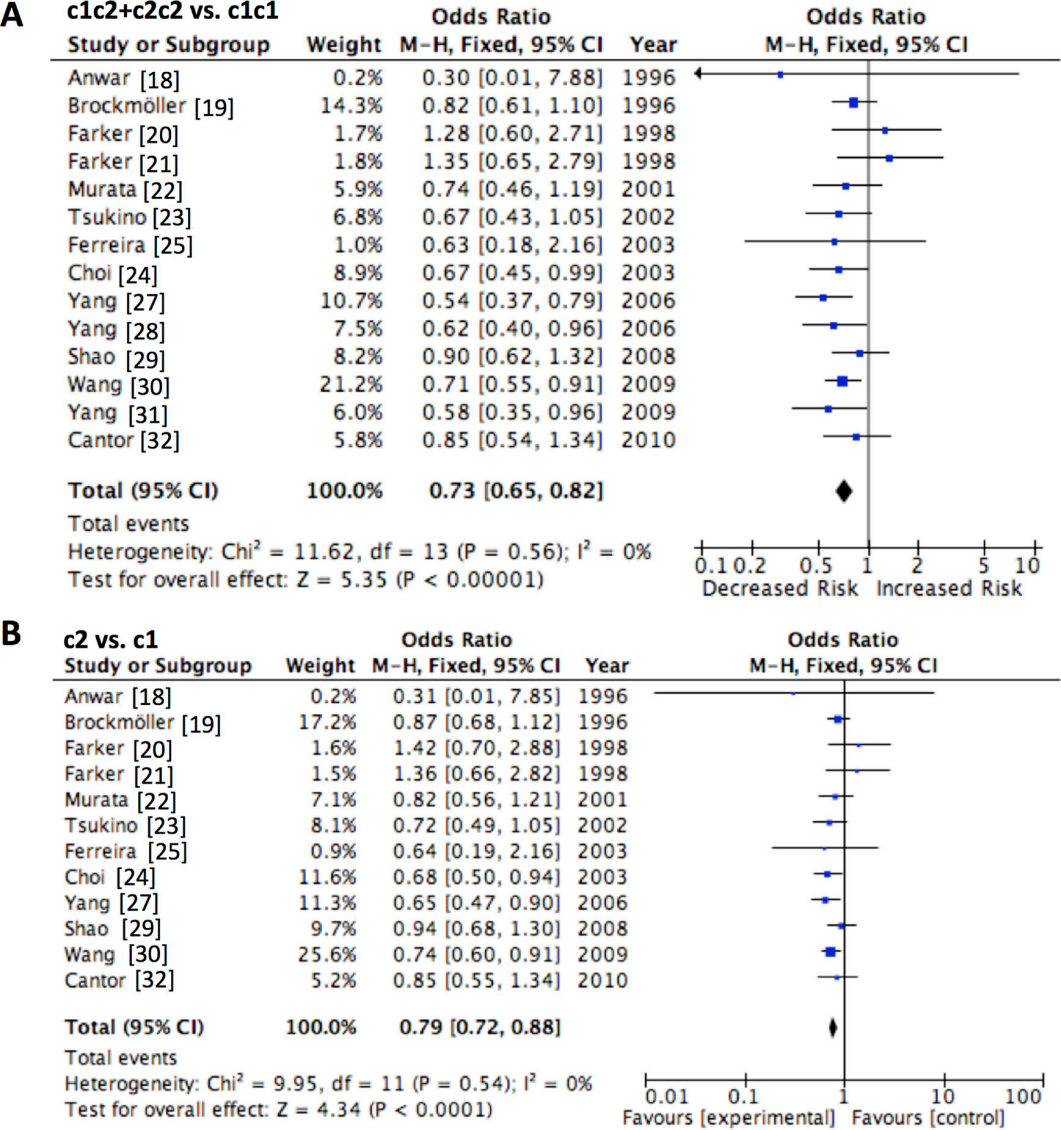
Forest plot of CYP2E1 Rsa I/Pst I polymorphism and the risk of urinary cancers in the (**A**) dominant and (**B**) allele models. Horizontal lines represent 95% CI. The area of each square represents the weighting and the positions of each square demonstrate the OR point estimate.

The main results about CYP2E1 RsaI/PstI polymorphism were summarized in Table 4. The association between CYP2E1 RsaI/PstI polymorphism and the susceptibility of urinary cancers was based upon 14 studies (Table 4, Figure 2). We observed a significant difference in the dominant model (c1c2 + c2c2 vs. c1c1, OR = 0.731, 95% CI = 0.681–0.790, *P <* 0.001) as well as the allele model (c2 vs. c1, OR = 0.793, 95% CI = 0.740–0.853, *P <* 0.001). Considering the ethnic discrepancy in the genotype/allele frequency of the polymorphism, we studied the impact of CYP2E1 RsaI/PstI in Asian and Caucasian population. A significant difference in c1c2+c2c2 vs. c1c1 and c2 vs. c1 among Asians was observed, with the summarized ORs being equal to 0.682 (95% CI = 0.601–0.784) and 0.751 (95% CI = 0.663–0.851) respectively. However, we failed to observe any difference among Caucasians in either dominant mode (OR = 0.889, 95% CI = 0.714–1.103, *P =* 0.281) or allele model (OR = 0.921, 95% CI = 0.762–1.120, *P =* 0.401). Summarized ORs for CYP2E1 RsaI/PstI stratified by cancer type were also evaluated. We observed subjects with c1c2/c2c2 genotype had decreased risk than those with c1c1 genotype for developing prostate, urothelial and bladder cancer, with the summarized ORs being equal to 0.609 (95% CI = 0.492–0.760, *P <* 0.001), 0.773 (95% CI = 0.633–0.941, *P =* 0.009) and 0.810 (95% CI = 0.672–0.970, *P =* 0.021) respectively. Consistently, similar results were observed with the allele model in the subgroup analysis considering cancer type. Subjects with the c2 allele had decreased susceptibility than those with the c1 allele for prostate, urothelial and bladder cancer, with the summarized ORs being 0.710 (95% CI = 0.562–0.910, *P =* 0.007), 0.794 (95% CI = 0.660–0.943, *P =* 0.007) and 0.832 (95% CI = 0.712–0.971, *P =* 0.020), respectively.

**Table 4:** Main results of meta-analysis for the association of CYP2E1 gene Rsa I/Pst I polymorphism and urinary cancers risk

Genetic Model	Groups/Subgroups	Studies, *n*	Heterogeneity Test		Statistical Model	Test for Overall Effect		
			I^2^, %	*P*		OR	95% CI	*P* value
c1c2+c2c2 vs. c1c1	Overall	14	0	0.671	Fixed	0.731	0.681–0.790	< 0.001
	Caucasian	6	0	0.672	Fixed	0.889	0.714–1.103	0.281
	Asian	8	0	0.738	Fixed	0.682	0.601–0.784	< 0.001
	Prostate	5	0	0.893	Fixed	0.609	0.492–0.760	< 0.001
	Urothelial	4	38	0.182	Fixed	0.773	0.633–0.941	0.009
	Bladder	5	0	0.798	Fixed	0.810	0.672–0.970	0.021
c2 vs. c1	Overall	12	0	0.649	Fixed	0.793	0.740–0.853	< 0.001
	Caucasian	6	0	0.612	Fixed	0.921	0.762–1.120	0.401
	Asian	6	0	0.657	Fixed	0.751	0.663–0.851	< 0.001
	Prostate	3	0	0.656	Fixed	0.710	0.562–0.910	0.007
	Urothelial	4	45	0.140	Fixed	0.794	0.660–0.943	0.007
	Bladder	5	0	0.643	Fixed	0.832	0.712–0.971	0.020

**Figure 3:**
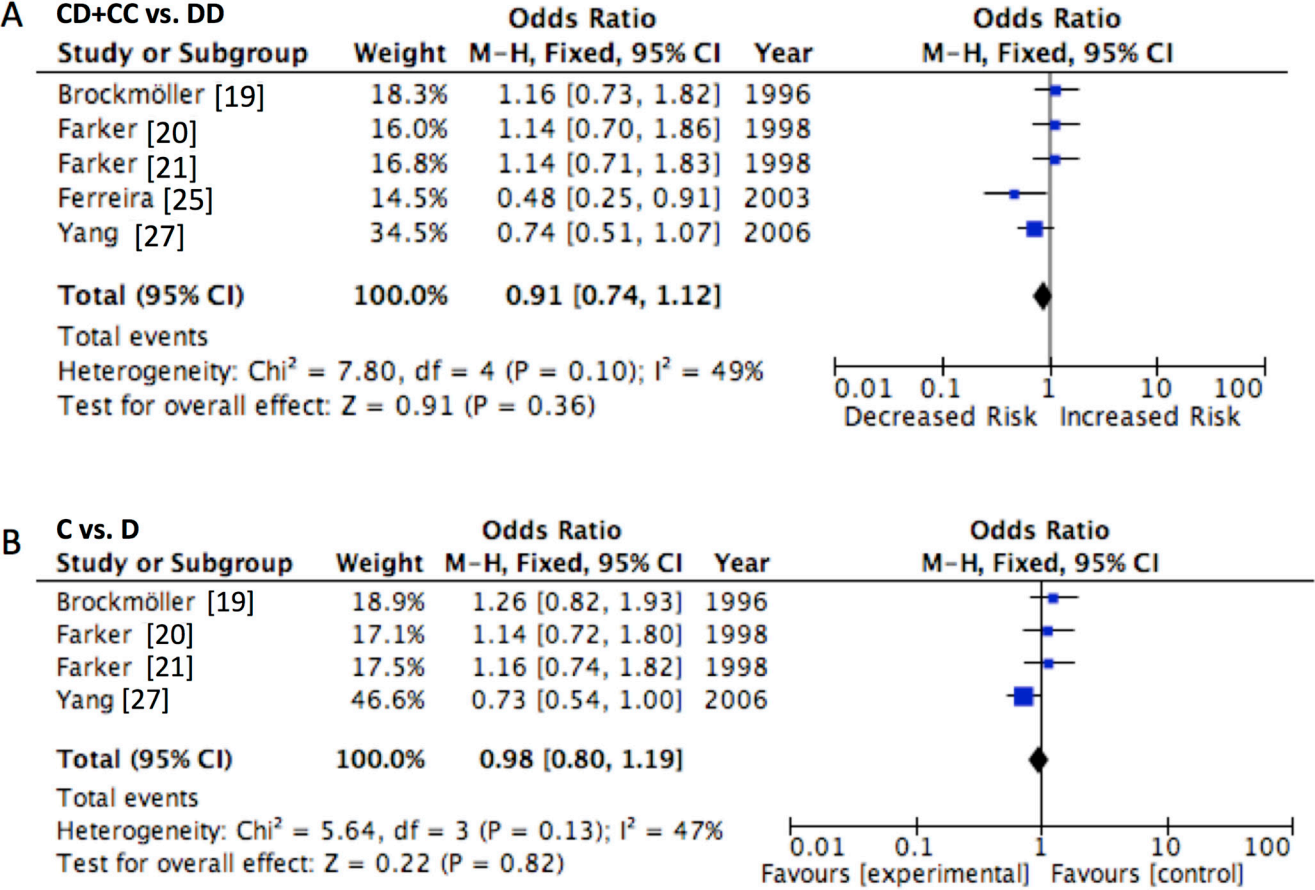
Forest plot of CYP2E1 Dra I polymorphism and the risk of urinary cancers in the (**A**) dominant and (**B**) allele models. Horizontal lines represent 95% CI. The area of each square represents the weighting and the positions of each square demonstrate the OR point estimate.

The principle results of CYP2E1 Dra I polymorphism were shown in Table 5. The effect of CYP2E1 Dra I polymorphism on the susceptibility of urinary cancers was analyzed on 5 case-control studies. However, no significant association between CYP2E1 Dra I polymorphism and urinary cancer susceptibility in either dominant model (CD+CC vs. DD, OR = 0.913, 95% CI = 0.791–1.051, *P =* 0.202) or the allele model (C vs. D, OR = 0.978, 95% CI = 0.847–1.117, *P* = 0.748) was showed. Further subgroup analysis considering ethnicity and cancer type was conducted. We demonstrated that prostate cancer patients carrying CD/CC genotype had 0.66-fold risk than patients with DD genotype (OR = 0.658, 95% CI = 0.479–0.907, P = 0.011). Other subgroup summarized ORs were found no significant difference (all *P* > 0.05) (Table 5, Figure 3).

**Table 5:** Main results of meta-analysis for the association of CYP2E1 gene Dra I polymorphism and urinary cancers risk

Genetic Model	Groups/Subgroups	Studies, n	Heterogeneity Test		Statistical Model	Test for Overall Effect		
			I^2^, %	*P*		OR	95% CI	*P* value
CD + CC vs. DD	Overall	5	42	0.102	Fixed	0.913	0.791–1.051	0.202
	Caucasian	4	49	0.112	Fixed	0.998	0.778–1.280	0.998
	Asian	1	NA			0.740	0.512–1.068	0.112
	Prostate	2	23	0.258	Fixed	0.658	0.479–0.907	0.011
	Urothelial	2	0	0.987	Fixed	1.141	0.809–1.601	0.439
	Bladder	1	NA			1.159	0.731–1.822	0.531
C vs. D	Overall	4	38	0.131	Fixed	0.978	0.847–1.117	0.748
	Caucasian	3	0	0.942	Fixed	1.192	0.920–1.541	0.181
	Asian	1	NA			0.730	0.544–1.013	0.052
	Prostate	1	NA			0.728	0.537–1.010	0.051
	Urothelial	2	0	0.970	Fixed	1.151	0.843–1.582	0.390
	Bladder	1	NA			1.259	0.820–1.933	0.282

NA: Not Applicable.

### Heterogeneity, sensitivity and bias diagnosis

In the meta-analysis, no significant heterogeneity was found in any of the comparisons (*P <* 0.10 or I^2^ ≥ 50%), and all the *P* values and I^2^ values were listed in Tables 4 and 5. One-way sensitivity analysis was conducted to assess the stability of this meta-analysis [37]. The statistical significance of the overall analyses did not vary even though any study was deleted from the overall data (data not shown), indicating the stability of the results.

**Figure 4:**
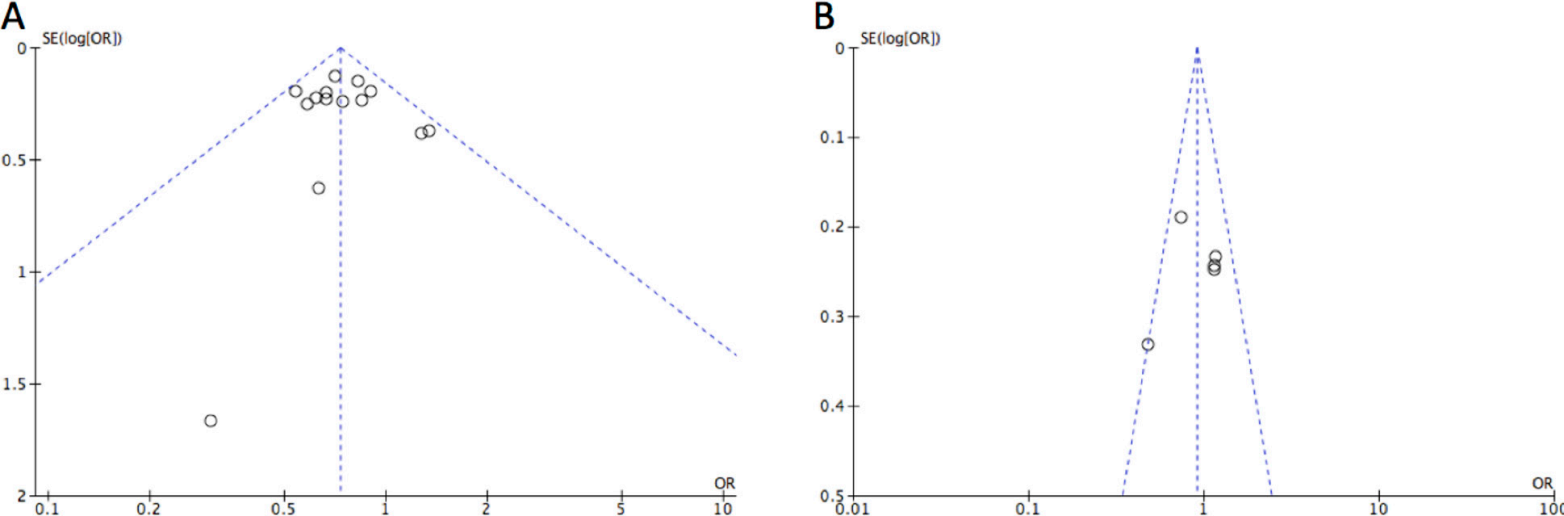
Funnel plot detect publication bias in the study for (**A**) CYP2E1Rsa I/Pst I and (**B**) Dra I polymorphisms in the dominant model. Each OR was reported on a log scale against its standard error (SE). The vertical line indicated the pooled estimate of the overall OR with the sloping lines representing the expected 95% CI for a given SE.

Publication bias was firstly examined by using funnel plot analysis (Figure 4). As a consequence, the funnel plot presented symmetrical shape of for the overall analysis; however, some uncertainty existed considering the symmetrical degrees were not satisfied. Therefore, the Begg's test was adopted to test the symmetry of funnel plot. Consistently, no publication bias was revealed considering the results of Begg’s test (*P* > 0.05).

## DISCUSSION

It is widely accepted that genetic susceptibility plays a key role in the development of urinary cancer, though the underlying mechanism of urinary cancer has not been clearly illuminated [4, 5]. A variety of genetic polymorphisms have been identified as risk factors for urinary cancer, some of which are found in the metabolism genes of carcinogens [38, 39]. CYP takes part in the oxidation of some chemicals and subsequently produce reactive free radicals which may lead to lipid peroxidation and carcinogenesis [40]. As a member of the CYP super family, CYP2E1 participates in the metabolism process of many carcinogens including N-nitrosamines and aniline. Therefore, it affects the susceptibility of host to urinary cancer [41, 42]. For instance, CYP2E1 takes part in the metabolic activation of kinds of N-nitrosamines, which are tobacco-related carcinogens of bladder in experimental animals. These enzymes can catalyze and activate the procarcinogens and ectogenous compounds in the beginning of metabolism, thereafter produce reactive metabolic intermediates and lead to genetic mutations [43]. Thus, CYP2E1 polymorphisms may have an impact on the development of urinary cancer.

According to previous reports, six restriction fragment length polymorphisms (RFLPs) were resides in CYP2E1 gene [6]. Amongst them, the RsaI/PstI polymorphism in the 5′-flanking region as well as the Dra I polymorphism in intron 6 may affect the susceptibility to urinary cancer based on their regulatory roles in CYP2E1 transcriptional activity [12, 15]. Numerous studies were conducted to assess the impact of RsaI/Pst I and Dra I polymorphism in the pathogenesis of urinary cancer; however, no consistent findings were reported. In the study, we conducted a meta-analysis of eligible studies to evaluate the association between CYP2E1 Rsa I/Pst I and Dra I polymorphisms and urinary cancer susceptibility.

As for CYP2E1 Rsa I/Pst I polymorphism, the results have demonstrated the mutated allele c2 may have different effects in different cancer type [43–46]. In recent two decades, several studies have been conducted to identify the relationship between CYP2E1 and urinary cancer with inconsistent conclusions [42]. Using 3,301 cases and 3,786 controls from 14 studies, we showed reduced urinary cancer risk in patients having CYP2E1 RsaI/PstI c1c2 + c2c2 genotype (OR = 0.73, 95% CI = 0.68–0.79, *P <* 0.001), comparing with the subjects carrying wide-type homozygous c1c1 genotype. In the subgroup analyses sorted by cancer category, significant associations were found in prostate cancer, urothelial cancer as well as bladder cancer group. Furthermore, ethnicity may influence CYP2E1 activity through gene–gene interactions since it is an important biological factor [6]. The allele frequency was significantly different between Asians and Caucasians, indicating the impact of ethnic diversity on the environment and genetic backgrounds. In consistence with the study by Wang et al. [6], c2 allele of CYP2E1 RsaI/PstI polymorphism is more common in Orientals compared to the Western population. In consequence, further meta-analysis stratified by ethnicity was conducted, and the decreased OR was found in the Asians for CYP2E1 Rsa I/Pst I polymorphisms (OR = 0.682, 95% CI = 0.601–0.784). However, we failed to observe any significant difference in the Caucasian populations, indicating that the risk in Asians is more obvious, which may be caused by different genetic backgrounds and environmental factors. However, the explicit reason accounting for the difference between Asians and Caucasians about gene-environment interactions is not clear now and need more studies to explore and deeper illustrate.

As for CYP2E1 Dra I polymorphism, we identified five studies that had examined the relationship between CYP2E1 Dra I polymorphism and urinary cancer. The pooled result failed to identified any significant associations between CYP2E1 Dra I polymorphism and the urinary cancer risk (CD + CC vs. DD, OR = 0.913, 95% CI = 0.791–1.051, *P =* 0.202; C vs. D, OR = 0.978, 95% CI = 0.847–1.117, *P* = 0.748). Further subgroup analysis sorted by the ethnicity and cancer category indicated that patients carrying CD/CC genotype had 0.658-fold risk compared with patients with DD genotype to develop into prostate cancer (OR = 0.658, 95% CI = 0.479–0.907, *P* = 0.011). Nevertheless, no significant association was found for Asians, Caucasians and other cancer types in neither genetic model. Since sample size is a key factor for identifying risk factors, we may undervalue the association between Dra I polymorphism and urinary cancer risk considering the limited quantity of eligible studies.

The relationship between clinical significance and these two polymorphisms has to be elucidated from the perspective of mechanism. CYP2E1 Rsa I/Pst I was mapped to the 5′-flanking region of the human CYP2E1 gene. According to Hayashi et al., the Chloramphenicol Acetyltransferase (CAT) assay demonstrated the enhancement of expression by c2c2, which was about 10 times than that by its c1c1 counterpart. The DNase I sensitivities and protection profiles of the two genotypes were also different, with RsaI polymorphism affecting the transcriptional activation of CYP2E1 as well as the binding capacity of transcription factor HNF-1, while the PstI polymorphism having no impact [12]. The study by Persson et al. also found RsaI polymorphism may affecting the binding of HNF-1 to the 5′-flanking region [47]. In addition, the impacts of different genotypes on the mRNA expression of CYP2E1 were examined in 86 individuals by Watanabe et al., with the alcohol consumption being considered. The expression of CYP2E1 mRNA in genotype c1c2 was 1.7-fold higher than that in genotype c1c1 in non-drinkers. While subjects with genotype c1c2 who drank alcohol were 2.0-fold higher than non-drinkers with genotype c1c1 [11], suggesting that RsaI polymorphism may result in individualized differences in CYP2E1 catalyzed oxidation activities. As for another polymorphism CYP2E1 DraI, the association between its allelic variant with the mRNA expression level was briefly reported. According Uematsu et al., the CYP2E1 Dra I polymorphism might have an impact on the mRNA expression of CYP2E1 [15]. However, these studies are not detailed enough to explain all the clinical significance. Since CYP2E1 are relatively well conserved without common functional polymorphisms [48], there exists another explanation that the polymorphisms might be in linkage disequilibrium to other polymorphisms that have impacts but the bases for any association to urinary cancer are presently obscure. Therefore, an adequate comprehension of the possible molecular factors and mechanisms underlying the observed results for urinary cancer is becoming an urgent priority.

This is the first meta-analysis assessing the association between CYP2E1 Rsa I/Pst I as well as Dra I polymorphisms and the susceptibility of urinary cancers. Nevertheless, when interpreting the results of the meta-analysis, limitations must be taken into account. Firstly, only five studies with 1,168 cases and 1,275 controls reported the association between CYP2E1 Dra I polymorphism and urinary cancer risk. Therefore, significant associations might be underpowered considering the limited studies involved. More studies with larger sample size are necessary to get a more precise conclusion. Secondly, owing to the relatively low frequency of mutated alleles of CYP2E1 polymorphism in Caucasians, we failed to distinguish any significant association in the Caucasians subgroup [44]. As a consequence, further studies evaluating the ethnic difference in the polymorphism on urinary cancer risk are in need. Thirdly, other important DMEs may act as potential confounding factors contributing to urinary cancers, particularly N-Acetyltransferases (NATs) which could detoxify aromatic amines, an important class of bladder carcinogens in tobacco smoke and implicate in urinary cancers [49] and Glutathione S-transferases (GSTs) which protect susceptible person due to occupational and environmental factor from reactive metabolites [50]. Both enzymes were reported to contribute to bladder cancer in other ethnic groups; thereby it may impact on our results as a potential confounding factor. Finally, the results applied in this meta-analysis were based on unadjusted estimates since we did not have access to most of the original data. More detailed analysis adjusted by sex, age or lifestyle should be conducted to estimate the associations of CYP2E1 Rsa I/Pst I and DraI polymorphisms with urinary cancer. Thus, it is required for the authors to share their data of all of the published papers.

In the present meta-analysis, we provided preliminarily genetic evidence that CYP2E1 Rsa I/Pst I polymorphism was related with the susceptibility of urinary cancers in Asian population, though Dra I polymorphism failed to contribute to the susceptibility of urinary cancers except for prostate cancer. However, our findings have to be interpreted with caution due to several limitations. Studies involved large number of participants and more homogeneous cancer patients should be future conducted.
